# Efficient Estimation Methods for the QR Distribution with Type-II Censored Data: An Empirical Validation on Lung Cancer Prognosis

**DOI:** 10.3390/e28050502

**Published:** 2026-04-29

**Authors:** Qasim Ramzan, Muhammad Amin, Shuhrah Alghamdi, Randa Alharbi

**Affiliations:** 1 Department of Statistics, Government Graduate College Jauharabad, Khushab 41200, Pakistan; qasimramzankbk@gmail.com; 2 Department of Statistics, University of Sargodha, Sargodha 40100, Pakistan; muhammad.amin@uos.edu.pk; 3 Department of Mathematical Sciences, Princess Nourah bint Abdulrahman University, Riyadh 11564, Saudi Arabia; 4 Department of Statistics, Faculty of Science, University of Tabuk, Tabuk 47512, Saudi Arabia; ralharbi@ut.edu.sa

**Keywords:** Bayesian estimation, lung cancer data, QR distribution, stochastic gradient descent, Type-II censoring, variational inference

## Abstract

The QR distribution, recently introduced for modeling lifetime data under Type-II censoring, offers a flexible framework for survival and reliability analysis. This study provides the first comprehensive evaluation of multiple modern estimation techniques for the QR distribution under Type-II censoring. We systematically compare classical maximum likelihood estimation with stochastic gradient descent variants (Momentum and Adam), Bayesian approaches including Maximum A Posteriori estimation, Markov Chain Monte Carlo, and Variational Inference, as well as machine learning-integrated methods such as amortized neural network inference. Using both synthetic and the real Veterans’ Administration Lung Cancer dataset, we evaluate these methods in terms of parameter estimation accuracy, computational efficiency, and convergence behavior. The results demonstrate the strengths of optimization-based, Bayesian, and neural approaches, highlighting their practical utility in handling complex censored survival data. This research validates the distribution’s effectiveness in capturing survival dynamics, offering valuable insights for clinical applications and highlighting areas for methodological improvement.

## 1. Introduction

Survival analysis and reliability modeling are fundamental in statistical research, particularly for applications in medical and engineering contexts where life-testing experiments often result in censored data. The continuous demand for highly adaptable probability models has driven significant advancements in distribution theory. Recently, ref. [1] introduced the QR distribution, providing an efficient framework for modeling complex survival times. However, to fully appreciate the mathematical architecture of the QR distribution, it is imperative to acknowledge its parent distributions within the Lindley family. The QR distribution is fundamentally an extended and exponentiated variant of the Lindley modeling framework. Specifically, by modifying the survival function of the Pseudo Lindley distribution proposed by [2], setting the parameter β=1, substituting θ with 2θ, and applying exponentiation, the foundational structure of the QR distribution is derived. Similarly, it can be viewed as a new exponentiated-Quasi Lindley distribution by applying equivalent parametric substitutions (α=0 and using 2θ instead of θ) to the Quasi Lindley model introduced by [3]. Recognizing this lineage ensures a robust theoretical grounding for our subsequent inferences.

In recent years, to establish distinct identities in the literature, it has become a standard academic practice to name novel distributions, control charts, or frameworks using specific acronyms or author-derived names. This convention traces back to foundational models like the Lindley distribution itself and continues to be widely adopted in contemporary distribution theory. For instance, recent literature features numerous robust models explicitly named after their developers or given designated monikers, such as the Shanker distribution [4], and the Ailamujia distribution [5]. In a similar vein, ref. [6] introduced the AMAZON framework for adaptive quantile monitoring, and ref. [7] developed the MARCONI control chart for monitoring Burr-X processes. The QR distribution proposed by [1] follows this contemporary naming convention while addressing specific gaps in modeling censored life data. Earlier work on censoring laid the foundational concepts for handling incomplete data. Ref. [8] introduced methods for analyzing progressively censored samples in life testing, establishing Type-II censoring as a practical approach for time-constrained experiments. Refs. [9,10] further developed the statistical properties and inference methods for progressive Type-II censoring. These seminal studies established a robust methodological framework for censored data that modern reliability studies, including our current analysis of the QR distribution, rely heavily upon to handle real-world scenarios like medical trials and industrial life testing.

Parametric distributions have historically evolved to capture increasingly complex failure rates. Ref. [11] proposed the exponentiated Weibull distribution to capture bathtub-shaped failure rates, a concept later extended by [12,13] through various modified Weibull models. Furthermore, ref. [14] introduced the beta-Pareto distribution, highlighting the necessity for statistical models that can accommodate diverse and asymmetric failure patterns. Later, ref. [15] proposed Burr XII-Weibull-logarithmic distribution. Rather than sharing direct hazard similarities, this historical progression demonstrates the continuous academic pursuit of flexible hazard structures. It focuses on developmental gap that Lindley-extended models like the QR distribution now aim to fill under complex censoring mechanisms. Recent developments in distribution theory have continued to introduce novel models specifically designed for survival data. Ref. [16] proposed the Sine distribution, deriving its properties via classical and Bayesian methods. Similarly, ref. [17] introduced the unit inverse Weibull G family, analyzing it under progressive Type-II censoring. Ref. [18] explored the Gumbel Type-II distribution under joint progressive Type-II censoring for multi-component systems. Furthermore, ref. [19] developed optimal weighted loss functions for Bayesian estimation of the Pareto model based on Type-II censoring. Ref. [20] compared Type-II half-logistic Weibull Cox-proportional hazard models, highlighting their capacity to capture non-proportional hazards.

Alongside theoretical development, estimation techniques for censored data have evolved significantly. Following [8]’s benchmark Maximum Likelihood Estimation (MLE) under censoring, refs. [21,22] extended MLE to progressively censored Gaussian and modified Weibull distributions. Bayesian methods later gained prominence for their ability to incorporate prior knowledge. Ref. [23] applied Bayesian inference to the exponentiated Weibull model under Type-II censoring. Refs. [24,25] further explored Bayesian estimation for modified Weibull and ridge parameters. Refs. [26,27] explored goodness-of-fit (GOF) and ranked set sampling for Burr Type X distributions, providing insights into robust estimation that informed our comparative methodologies. To overcome the challenges of complex likelihood surfaces, stochastic optimization and machine learning techniques have emerged as powerful tools. Ref. [28] introduced hybrid Monte Carlo methods, inspiring modern gradient-based algorithms. Ref. [29] successfully applied stochastic gradient descent (SGD) to the alpha power Weibull distribution under progressive censoring. More recently, literature has emphasized neural network (NN)-based and Approximate Bayesian Computation (ABC) approaches for scalable inference. Ref. [30] investigated transmuted distributions with NN applications, and ref. [31] applied the Marshall-Olkin-Weibull logarithmic distribution to censored clinical data. Additionally, ref. [32] employed Bayesian methods for mixture distributions in fatigue fracture data.

Despite these advancements, a significant gap remains in integrating Lindley-based models with modern, scalable machine learning estimation frameworks under censoring. The primary novelty of this research lies in comprehensively bridging this gap. The study builds directly upon the foundational work of [1], who proposed the QR distribution specifically for modeling survival times under Type-II censoring and demonstrated its robustness through extensive simulations. While ref. [1] focused primarily on classical and basic Bayesian estimation, our work advances the literature by providing the first comprehensive comparative evaluation of multiple modern estimation techniques for the QR distribution under Type-II censoring. This study offers a multi-faceted computational framework, integrating SGD with momentum, Adam optimization, Markov Chain Monte Carlo (MCMC), Variational Inference (VI), and NN-based ABC, applied to the QR distribution under Type-II censoring. By systematically comparing traditional classical methods with these cutting-edge AI-driven approaches on both synthetic setups and the Lung Cancer datasets, this study establishes a new benchmark for scalable, and parameter estimation in reliability engineering. This approach offers practical guidance on method selection for researchers dealing with censored survival data in clinical settings.

Despite the robust methodological contributions, this study has certain limitations that must be acknowledged. First, the experimental framework relies heavily on Type-II censoring; the performance of the proposed advanced estimators (such as ABC-MDN and VI) under more complex schemes like adaptive, progressive or interval censoring remains unexplored. Second, while the SGD, and ABC approaches offer exceptional flexibility, they introduces significant computational overhead during the training phase compared to standard MLE. Finally, while validated against clinical data, broader application to highly dynamic industrial engineering datasets with time-varying covariates is required to fully confirm the real-world generalization of the proposed algorithms. These limitations highlight promising directions for future research, including broader simulation studies under various censoring mechanisms and applications to multi-component systems.

The remainder of this paper is organized as follows: Section 2 details the model and parameter estimation, exploring various classical and modern estimation techniques for the QR distribution. Section 4 presents the interval estimation, focusing on methods to quantify parameter uncertainty. Concluding remarks and potential extensions are discussed in Section 6.

## 2. The Model and Estimation Methods

This section outlines the methods employed to estimate the parameters of the QR distribution, leveraging both classical and advanced computational approaches to ensure robust modeling of survival data under Type-II censoring. The QR distribution introduced by [1] with its cumulative distribution function (CDF) is defined by

(1) $$\begin{array}{ccc} F_{QR}\left(x\right) & = & \gamma \int_{0}^{-\log\left[\left(1+2\theta x\right)e^{-2\theta x}\right]}e^{-\gamma t}dt.\\ & = & 1-{\left(1+2\theta x\right)}^{\gamma }e^{-2\theta \gamma x}\;x\geq 0, \end{array}$$

where θ,γ>0 are respectively the scale and shape parameters. The probability distribution function (PDF) corresponding to (1) is given by

(2) $$\begin{array}{ccc} f_{QR}\left(x\right) & = & 4\theta^{2}\gamma x{\left(1+2\theta x\right)}^{\gamma -1}e^{-2\theta \gamma x}. \end{array}$$

**Figure 1:**
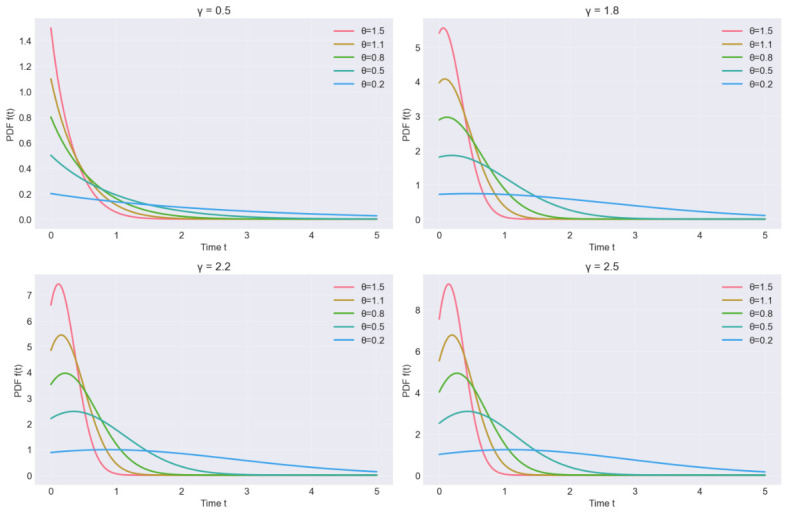
PDF plot of the QR distribution for selected values of θ and γ.

**Figure 2:**
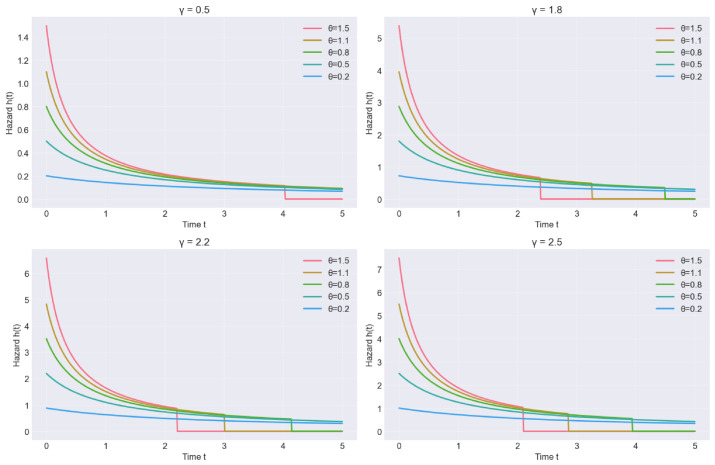
Hazard Rate Function (HRF) plot of the QR distribution for selected values of θ and γ.

To illustrate the flexibility of the proposed QR distribution, Figure 1 and Figure 2 show the PDF and hazard rate functions for different parameter values. The hazard function exhibits increasing, decreasing, or unimodal behaviors depending on θ and γ, making the QR distribution suitable for various reliability scenarios.

### Maximum Likelihood Estimation with Stochastic Gradient Descent

The SGD is an optimization algorithm from machine learning used to minimize the negative log-likelihood for parameter estimation in statistical models. The SGD iteratively updates parameters θ and γ using mini-batches of data to approximate the gradient, making it efficient for large datasets. This is an advanced estimation method alternative to Newton-Raphson, incorporating adaptive learning rates (e.g., Adam variant) for faster convergence.

The log-likelihood for the QR model under Type-II censored data is:

(3) $$\begin{array}{ccc} l(\theta ,\gamma |X) & = & \log C+2m\log\theta +m\log\gamma +\sum_{i=1}^{m}\log x_{i}+\left(\gamma -1\right)\sum_{i=1}^{m}\log\left(1+2\theta x_{i}\right)\\ & & +\gamma \left(n-m\right)\log\left(1+2\theta x_{m}\right)-2\theta \gamma \left\{\sum_{i=1}^{m}x_{i}+x_{m}\left(n-m\right)\right\}, \end{array}$$

where C=n!/(n−m)!, and $X=\{x_{1},\ldots ,x_{m}\}$ are the ordered failure times.

To apply SGD, minimize the negative log-likelihood L(θ,γ)=−l(θ,γ∣X). The gradients are derived from partial derivatives concerning γ and θ as

$$\frac{\partial l}{\partial \gamma }=\frac{m}{\gamma }+\sum_{i=1}^{m}\log\left(1+2\theta x_{i}\right)+\left(n-m\right)\log\left(1+2\theta x_{m}\right)-2\theta \left\{\sum_{i=1}^{m}x_{i}+x_{m}\left(n-m\right)\right\},$$

$$\frac{\partial l}{\partial \theta }=\frac{2m}{\theta }+2\left(\gamma -1\right)\sum_{i=1}^{m}\frac{x_{i}}{1+2\theta x_{i}}+\frac{2\gamma \left(n-m\right)x_{m}}{1+2\theta x_{m}}-2\gamma \left\{\sum_{i=1}^{m}x_{i}+x_{m}\left(n-m\right)\right\}.$$

Thus,

$$\nabla_{\gamma }L=-\frac{\partial l}{\partial \gamma },\;\nabla_{\theta }L=-\frac{\partial l}{\partial \theta }.$$

In SGD, initialize parameters $\theta^{\left(0\right)},\gamma^{\left(0\right)}$. For each iteration k=1,2,…,K, sample a mini-batch B⊆X of size b≪m, compute approximate gradients ${\hat{\nabla }}_{\gamma }L$ and ${\hat{\nabla }}_{\theta }L$ over B, and update:

$$\theta^{\left(k\right)}=\theta^{\left(k-1\right)}-\eta {\hat{\nabla }}_{\theta }L,\;\gamma^{\left(k\right)}=\gamma^{\left(k-1\right)}-\eta {\hat{\nabla }}_{\gamma }L,$$

where η is the learning rate (e.g., decaying as $\eta_{k}=\eta_{0}/k$). For momentum SGD, introduce velocity terms:

$$v_{\theta }^{\left(k\right)}=\mu v_{\theta }^{\left(k-1\right)}-\eta {\hat{\nabla }}_{\theta }L,\;\theta^{\left(k\right)}=\theta^{\left(k-1\right)}+v_{\theta }^{\left(k\right)},$$

similarly for γ, with momentum μ≈0.9.

Convergence is monitored via L on a validation set or early stopping. This method handles non-convexity in the log-likelihood of the QR model and is better than Newton-Raphson by escaping local minima through stochasticity. Algorithm 1 outlines the iterative MLE process utilizing SGD inference methodology for parameter estimation. Where, epoch refers to one complete iteration through the dataset (or a full batch update cycle in stochastic gradient descent). During each epoch, the parameters are updated based on the computed gradients of the objective function (negative log-likelihood or negative log-posterior). The maximum number of epochs ($epochs_{m}$) is chosen empirically by monitoring the convergence behavior of the loss function (or log-posterior value). Training is terminated either when the loss stabilizes or when an early-stopping criterion is met (no significant improvement for a predefined number of epochs). This approach balances computational efficiency with reliable convergence, as demonstrated in the convergence diagnostics throughout the study.
**Algorithm 1** MLE with Stochastic Gradient Descent Variants.Initialize $\theta^{\left(0\right)},\gamma^{\left(0\right)}$**for** epoch = 1 to $epochs_{m}$ **do**      Compute gradient of negative log-likelihood w.r.t. θ,&γ      Update parameters using Adam/Momentum rule**end for****return** $\hat{\theta },\hat{\gamma }$

## 3. Bayesian Estimation Framework

Bayesian estimation provides a natural framework for incorporating prior information and quantifying uncertainty in the parameters of the QR distribution under Type-II censoring. In this subsection, we consider three complementary Bayesian approaches: Markov Chain Monte Carlo (MCMC) sampling, Maximum A Posteriori (MAP) estimation, and Variational Inference (VI). These methods allow us to obtain both point estimates and full posterior distributions, offering richer inferential insights compared to classical approaches. For these analysis, the Bayesian point estimates were calculated as the posterior mean. In Bayesian decision theory, the posterior mean is the optimal estimator that minimizes the Squared Error Loss Function (SELF), defined as $L\left(\hat{\delta },\delta \right)={\left(\hat{\delta }-\delta \right)}^{2}$. The posterior is proportional to the product of the Type-II censored likelihood and the prior distributions (independent Gamma priors on θ and γ). This choice ensures that our estimates are both unbiased relative to the posterior and minimize the resulting risk.

### 3.1. Markov Chain Monte Carlo Approach

The MCMC sampling technique (e.g., via Metropolis-Hastings or Gibbs), estimates the parameters of posterior distribution. For the QR distribution under Type-II censoring, assume priors for θ and γ are respectively given as $\pi \left(\theta \right)∼\mathrm{Gamma}\left(\alpha_{\theta },\beta_{\theta }\right)$ and $\pi \left(\gamma \right)∼\mathrm{Gamma}\left(\alpha_{\gamma },\beta_{\gamma }\right)$ for conjugacy-like properties.

The PDF of the posterior distribution is:

p(θ,γ∣X)∝L(θ,γ∣X)π(θ)π(γ),

where *L* is the likelihood. The joint posterior distribution under QR settings becomes:

(4) $$\begin{array}{ccc} p(\gamma ,\theta |X) & \propto & \theta^{2m+a-1}\gamma^{m+\ddot{a}-1}{\left(1+2\theta x_{m}\right)}^{\gamma (n-m)}e^{-\left[2\theta \gamma \left\{\sum_{i=1}^{m}x_{i}+x_{m}\left(n-m\right)\right\}+\gamma \ddot{b}+\theta b\right]}\prod_{i=1}^{m}x_{i}{\left(1+2\theta x_{i}\right)}^{\gamma -1}. \end{array}$$

The joint log-posterior is:

(5) $$\begin{array}{ccc} \log p(\theta ,\gamma |X) & = & l\left(\theta ,\gamma |X\right)+\left(\alpha_{\theta }-1\right)\log\theta -\beta_{\theta }\theta +\left(\alpha_{\gamma }-1\right)\log\gamma -\beta_{\gamma }\gamma +\mathrm{const}.\\ & = & \left(2m+a-1\right)\log\theta +\left(m+\ddot{a}-1\right)\log\gamma +\gamma \left(n-m\right)\log\left(1+2\theta x_{m}\right)\\ & & -\left[2\theta \gamma \left\{\sum_{i=1}^{m}x_{i}+x_{m}\left(n-m\right)\right\}+\gamma \ddot{b}+\theta b\right]+\sum_{i=1}^{m}\log x_{i}\\ & & +\left(\gamma -1\right)\sum_{i=1}^{m}\log\left(1+2\theta x_{i}\right)+\mathrm{const}. \end{array}$$

Using Gibbs sampling, alternate sampling from conditionals. The conditional density for γ is given as:

$$p\left(\gamma |\theta ,X\right)\propto \gamma^{m+\alpha_{\gamma }-1}\exp\left(-\gamma \left[2\theta \left\{\sum_{i=1}^{m}x_{i}+x_{m}\left(n-m\right)\right\}+\beta_{\gamma }\right]\right)\prod_{i=1}^{m}{\left(1+2\theta x_{i}\right)}^{\gamma -1}{\left(1+2\theta x_{m}\right)}^{\gamma (n-m)}.$$

Which simplifies to a non-standard form, requiring Metropolis-Hastings within Gibbs. Propose $\gamma^{\prime }∼N\left(\gamma ,\sigma_{\gamma }^{2}\right)$, accept with probability:

$$\alpha \left(\gamma ,\gamma^{\prime }\right)=\min\left(1,\frac{p\left(\gamma^{\prime }|\theta ,X\right)q\left(\gamma |\gamma^{\prime }\right)}{p\left(\gamma |\theta ,X\right)q\left(\gamma^{\prime }|\gamma \right)}\right),$$

where *q* is the proposal density.

Similarly for θ:

$$p\left(\theta |\gamma ,X\right)\propto \theta^{2m+\alpha_{\theta }-1}\exp\left(-\theta \left[2\gamma \left\{\sum_{i=1}^{m}x_{i}+x_{m}\left(n-m\right)\right\}+\beta_{\theta }\right]\right)\prod_{i=1}^{m}{\left(1+2\theta x_{i}\right)}^{\gamma -1}{\left(1+2\theta x_{m}\right)}^{\gamma (n-m)}.$$

### 3.2. Bayesian Stochastic Gradient Descent

In the Bayesian framework, the SGD can be adapted to optimize the posterior mode or sample from the posterior via stochastic variational methods. Here, we derive the SGD for MAP estimation under the given priors. To find MAP estimates, maximize logp in Equation (5) using SGD. The gradients are:

(6) $$\begin{array}{ccc} \frac{\partial \log p}{\partial \gamma } & = & \frac{m+\ddot{a}-1}{\gamma }+\left(n-m\right)\log\left(1+2\theta x_{m}\right)+\sum_{i=1}^{m}\log\left(1+2\theta x_{i}\right)\\ & & -\left[2\theta \left\{\sum_{i=1}^{m}x_{i}+x_{m}\left(n-m\right)\right\}+\ddot{b}\right], \end{array}$$

(7) $$\begin{array}{ccc} \frac{\partial \log p}{\partial \theta } & = & \frac{2m+a-1}{\theta }+2\left(\gamma -1\right)\sum_{i=1}^{m}\frac{x_{i}}{1+2\theta x_{i}}+\frac{2\gamma \left(n-m\right)x_{m}}{1+2\theta x_{m}}\\ & & -\left[2\gamma \left\{\sum_{i=1}^{m}x_{i}+x_{m}\left(n-m\right)\right\}+b\right]. \end{array}$$

For SGD, minimize −logp. Initialize $\theta^{\left(0\right)},\gamma^{\left(0\right)}$. For iteration *k*, using mini-batch B:

(8) $$\begin{array}{ccc} {\hat{\theta }}^{\left(k\right)} & = & {\hat{\theta }}^{\left(k-1\right)}+\eta \frac{\partial \log p}{\partial \theta }{|}_{B},\;{\hat{\gamma }}^{\left(k\right)}={\hat{\gamma }}^{\left(k-1\right)}+\eta \frac{\partial \log p}{\partial \gamma }{|}_{B}. \end{array}$$

Algorithm 2 details the posterior sampling procedure employing the No-U-Turn Sampler (NUTS) with assigned Gamma priors.
**Algorithm 2** Bayesian MCMC (NUTS Sampler).Define prior: $\theta ∼\mathrm{Gamma}(a_{1},b_{1})$, $\gamma ∼\mathrm{Gamma}(a_{2},b_{2})$Define likelihood: L(t,e|θ,γ)Target posterior ∝ likelihood × priorRun NUTS sampler for *N* iterations with *M* chains**return** Posterior samples ${\left\{\theta^{\left(i\right)},\gamma^{\left(i\right)}\right\}}_{i=1}^{N}$

### 3.3. VI Based Approximate Bayesian Estimation

The VI, a deep learning optimization technique, approximates the posterior p(θ,γ∣X) with a variational distribution q(ϕ), minimizing Kullback-Leibler (KL) divergence. For the QR distribution, assume mean-field $q\left(\theta ,\gamma \right)=q_{\theta }\left(\theta \right)q_{\gamma }\left(\gamma \right)$, with $q_{\theta }∼\log N\left(\mu_{\theta },\sigma_{\theta }^{2}\right)$, $q_{\gamma }∼\log N\left(\mu_{\gamma },\sigma_{\gamma }^{2}\right)$, parameters $\phi =\{\mu_{\theta },\sigma_{\theta },\mu_{\gamma },\sigma_{\gamma }\}$.

The Evidence Lower Bound (ELBO) for the QR distribution is:

(9) $$\mathrm{ELBO}\left(\phi \right)=E_{q}\left[\log p\left(\theta ,\gamma ,X\right)\right]-E_{q}\left[\log q\left(\theta ,\gamma \right)\right],$$

where logp(θ,γ,X)=l(θ,γ∣X)+logπ(θ)+logπ(γ).

Using reparameterization trick for sampling: $\theta =\mu_{\theta }+\sigma_{\theta }\epsilon_{\theta }$, $\epsilon_{\theta }∼N\left(0,1\right)$; similarly for γ. Approximate expectations with Monte Carlo simulation:

$$\hat{\mathrm{ELBO}}\left(\phi \right)=\frac{1}{S}\sum_{s=1}^{S}\left[l\left(\theta^{\left(s\right)},\gamma^{\left(s\right)}|X\right)+\log\pi \left(\theta^{\left(s\right)}\right)+\log\pi \left(\gamma^{\left(s\right)}\right)-\log q_{\theta }\left(\theta^{\left(s\right)}\right)-\log q_{\gamma }\left(\gamma^{\left(s\right)}\right)\right].$$

Optimize ϕ via gradient ascent: $\phi \leftarrow \phi +\eta \nabla_{\phi }\hat{\mathrm{ELBO}}$, using Adam optimizer. To derive the explicit gradients for the ELBO in the VI setup, we start with the log joint posterior and the variational family. The derivations are based on the reparameterization trick for log-normal distributions, ensuring differentiability. We use symbolic computation to verify and simplify the expressions. The partial derivatives of the log posterior are taken form (6) and (7). The VI approximates the joint posterior f(γ,θ∣X) in Equation (4) by optimizing a variational distribution q(γ,θ) to minimize the KL divergence:

(10) $$\mathrm{KL}\left(q∥f\right)=E_{q}\left[\log\frac{q(\gamma ,\theta )}{f(\gamma ,\theta |X)}\right].$$

This is equivalent to maximizing the ELBO in Equation (9). For the QR distribution, assume a mean-field variational family:

$$q\left(\gamma ,\theta \right)=q_{\gamma }\left(\gamma \right)q_{\theta }\left(\theta \right),$$

where $q_{\gamma }\left(\gamma \right)∼\log N\left(\mu_{\gamma },\sigma_{\gamma }^{2}\right)$ and $q_{\theta }\left(\theta \right)∼\log N\left(\mu_{\theta },\sigma_{\theta }^{2}\right)$, chosen for positivity of parameters. The variational parameters are $\phi =\{\mu_{\gamma },\sigma_{\gamma },\mu_{\theta },\sigma_{\theta }\}$.

The entropy term is:

$$-E_{q}\left[\log q\left(\gamma ,\theta \right)\right]=\frac{1}{2}\log\left(2\pi e\sigma_{\gamma }^{2}\right)+\frac{1}{2}\log\left(2\pi e\sigma_{\theta }^{2}\right).$$

To compute $E_{q}\left[\log p\right]$, use reparameterization for differentiable sampling. Let $\gamma =\exp(\mu_{\gamma }+\sigma_{\gamma }\epsilon_{\gamma })$, $\theta =\exp(\mu_{\theta }+\sigma_{\theta }\epsilon_{\theta })$, with $\epsilon_{\gamma },\epsilon_{\theta }∼N\left(0,1\right)$. Approximate with Monte Carlo (*S* samples):

(11) $$E_{q}\left[\log p\right]\approx \frac{1}{S}\sum_{s=1}^{S}\log p\left(\gamma^{\left(s\right)},\theta^{\left(s\right)}|X\right).$$

Gradients of ELBO w.r.t. ϕ are computed via auto-differentiation on terms like:

(12) $$\frac{\partial }{\partial \mu_{\phi }}\mathrm{ELBO}\approx \frac{1}{S}\sum_{s=1}^{S}\left(\frac{\partial \log p}{\partial \phi^{\left(s\right)}}\cdot \sigma_{\phi }\epsilon_{\phi }^{\left(s\right)}-1\right).$$

Thus, the gradient of $E_{q}\left[\log p\right]$ w.r.t. $\mu_{\gamma }$ is $E\left[\frac{\partial \log p}{\partial \gamma }\gamma \right]$, and w.r.t. $\sigma_{\gamma }$ is $E\left[\frac{\partial \log p}{\partial \gamma }\gamma \epsilon_{\gamma }\right]$, and can be derived as:

(13) $$\begin{array}{ccc} \frac{\partial \mathrm{ELBO}}{\partial \mu_{\gamma }} & = & E\left[\left(\frac{m+\ddot{a}-1}{\gamma }+\left(n-m\right)\log\left(1+2\theta x_{m}\right)+\sum_{i=1}^{m}\log\left(1+2\theta x_{i}\right)-2\theta S-\ddot{b}\right)\gamma \right]+1\\ & = & E\left[m+\ddot{a}-1+\gamma \left(\left(n-m\right)\log\left(1+2\theta x_{m}\right)+\sum_{i=1}^{m}\log\left(1+2\theta x_{i}\right)-2\theta S-\ddot{b}\right)\right]+1, \end{array}$$

(14) $$\begin{array}{ccc} \frac{\partial \mathrm{ELBO}}{\partial \sigma_{\gamma }} & = & E\left[\left(\frac{m+\ddot{a}-1}{\gamma }+\left(n-m\right)\log\left(1+2\theta x_{m}\right)+\sum_{i=1}^{m}\log\left(1+2\theta x_{i}\right)-2\theta S-\ddot{b}\right)\gamma \epsilon_{\gamma }\right]+\frac{1}{\sigma_{\gamma }}. \end{array}$$

Similarly for θ:

(15) $$\begin{array}{ccc} \frac{\partial \mathrm{ELBO}}{\partial \mu_{\theta }} & = & E\left[\left(\frac{2m+a-1}{\theta }+\frac{2\gamma \left(n-m\right)x_{m}}{1+2\theta x_{m}}+2\left(\gamma -1\right)\sum_{i=1}^{m}\frac{x_{i}}{1+2\theta x_{i}}-2\gamma S-b\right)\theta \right]+1\\ & = & E\left[2m+a-1+\theta \left(\frac{2\gamma \left(n-m\right)x_{m}}{1+2\theta x_{m}}+2\left(\gamma -1\right)\sum_{i=1}^{m}\frac{x_{i}}{1+2\theta x_{i}}-2\gamma S-b\right)\right]+1, \end{array}$$

(16) $$\begin{array}{ccc} \frac{\partial \mathrm{ELBO}}{\partial \sigma_{\theta }} & = & E\left[\left(\frac{2m+a-1}{\theta }+\frac{2\gamma \left(n-m\right)x_{m}}{1+2\theta x_{m}}+2\left(\gamma -1\right)\sum_{i=1}^{m}\frac{x_{i}}{1+2\theta x_{i}}-2\gamma S-b\right)\theta \epsilon_{\theta }\right]+\frac{1}{\sigma_{\theta }}. \end{array}$$

Optimize ϕ in Equation (12) using Adam: $\phi^{\left(k\right)}=\phi^{\left(k-1\right)}+\eta \nabla_{\phi }\mathrm{ELBO}$. Post-optimization, posterior means are $\hat{\theta }=\exp\left(\mu_{\theta }+\sigma_{\theta }^{2}/2\right)$, $\hat{\gamma }=\exp\left(\mu_{\gamma }+\sigma_{\gamma }^{2}/2\right)$. Post-convergence, approximate posterior expectations for ν(γ,θ). Sample $S^{\prime }$ from *q*:

$${\hat{\nu }}_{B}=\frac{1}{S^{\prime }}\sum_{s=1}^{S^{\prime }}\nu \left(\gamma^{\left(s\right)},\theta^{\left(s\right)}\right).$$

### 3.4. Neural Network Amortized Inference

Amortized inference uses a NN $g_{\phi }\left(X;\phi \right)$ to learn a mapping from data to parameter estimates, trained on simulated QR datasets. This deep learning approach is efficient for repeated estimations. The network structure includes an input layer for vectorized data X, hidden layers with ReLU activation, and an output layer that predicts $\hat{\theta }$ and $\hat{\gamma }$. Training data is generated by simulating *D* datasets ${\left\{X^{\left(d\right)}\right\}}_{d=1}^{D}$ from QR distribution with random $\theta ∼U(\theta_{\min},\theta_{\max})$, $\gamma ∼U(\gamma_{\min},\gamma_{\max})$, then applying Type-II censoring.

The model is trained by minimizing mean squared error (MSE):

$$L\left(\phi \right)=\frac{1}{D}\sum_{d=1}^{D}\left[{\left(\theta^{\left(d\right)}-g_{\phi ,\theta }\left(X^{\left(d\right)}\right)\right)}^{2}+{\left(\gamma^{\left(d\right)}-g_{\phi ,\gamma }\left(X^{\left(d\right)}\right)\right)}^{2}\right],$$

or alternatively using negative log-likelihood for probabilistic outputs. Parameters ϕ are updated through backpropagation with optimizers $\phi \leftarrow \phi -\eta \nabla_{\phi }L$, using SGD/Adam.

The NN input is summary statistics $s\left(X\right)=\{\sum x_{i},\sum \log\left(1+2\theta x_{i}\right),\ldots \}$ or raw X (padded). Architecture: Input layer, hidden layers with ReLU:

$$h_{1}=\mathrm{relu}\left(W_{1}s\left(X\right)+b_{1}\right),\;h_{l}=\mathrm{relu}\left(W_{l}h_{l-1}+b_{l}\right),$$

output $\hat{\phi }=W_{L}h_{L-1}+b_{L}$, where $\hat{\phi }$ are estimated $\mu_{\gamma },\sigma_{\gamma },\mu_{\theta },\sigma_{\theta }$.

Training minimizes the negative ELBO over simulations:

$$L\left(\phi \right)=-\frac{1}{D}\sum_{d=1}^{D}\mathrm{ELBO}\left(q_{{\hat{\phi }}^{\left(d\right)}};\gamma^{\left(d\right)},\theta^{\left(d\right)},X^{\left(d\right)}\right),$$

where ELBO uses the simulated true parameters for supervision.

Gradients for optimization are:

$$\nabla_{\phi }L=-\frac{1}{D}\sum_{d=1}^{D}\nabla_{\phi }\left(E_{q_{{\hat{\phi }}^{\left(d\right)}}}\left[\log p\left(\gamma ,\theta |X^{\left(d\right)}\right)\right]-E_{q_{{\hat{\phi }}^{\left(d\right)}}}\left[\log q_{{\hat{\phi }}^{\left(d\right)}}\left(\gamma ,\theta \right)\right]\right),$$

computed via backprop.

For inference on new X, compute $\hat{\phi }=g_{\phi }\left(X\right)$, then sample from $q_{\hat{\phi }}$ to get expectations as in VI.

For direct prediction of moments, train to minimize:

$$L\left(\phi \right)=\frac{1}{D}\sum_{d=1}^{D}\left(E\left[\gamma^{\left(d\right)}\right]-g_{\phi ,\gamma }{\left(X^{\left(d\right)}\right)}^{2}+E\left[\theta^{\left(d\right)}\right]-g_{\phi ,\theta }{\left(X^{\left(d\right)}\right)}^{2}\right),$$

adjusting for loss functions by training on transformed targets (e.g., $\nu^{-k_{1}}$ for General Entropy). In amortized inference, the NN $g_{\phi }\left(s\left(X\right)\right)$ outputs variational parameters ${\hat{\mu }}_{\gamma },{\hat{\sigma }}_{\gamma },{\hat{\mu }}_{\theta },{\hat{\sigma }}_{\theta }$. The training loss is the negative average ELBO over simulated datasets:

(17) $$\begin{array}{ccc} L(\phi ) & = & -\frac{1}{D}\sum_{d=1}^{D}\mathrm{ELBO}\left(q_{{\hat{\phi }}^{\left(d\right)}}|X^{\left(d\right)}\right)\\ & = & -\frac{1}{D}\sum_{d=1}^{D}\left(E_{q_{{\hat{\phi }}^{\left(d\right)}}}\left[\log p\left(\gamma ,\theta |X^{\left(d\right)}\right)\right]+\mathrm{entropy}\left(q_{{\hat{\phi }}^{\left(d\right)}}\right)\right). \end{array}$$

The gradient w.r.t. ϕ is:

(18) $$\begin{array}{ccc} \nabla_{\phi }L & = & -\frac{1}{D}\sum_{d=1}^{D}\nabla_{\phi }{\mathrm{ELBO}}^{\left(d\right)}=-\frac{1}{D}\sum_{d=1}^{D}\left(\nabla_{\hat{\phi }}{\mathrm{ELBO}}^{\left(d\right)}\cdot \nabla_{\phi }{\hat{\phi }}^{\left(d\right)}\right), \end{array}$$

where $\nabla_{\hat{\phi }}\mathrm{ELBO}$ uses the ELBO gradients derived above, and $\nabla_{\phi }{\hat{\phi }}^{\left(d\right)}=\nabla_{\phi }g_{\phi }\left(s\left(X^{\left(d\right)}\right)\right)$ is computed via backpropagation through the network layers.

For a feedforward NN with layers $h_{l}=\sigma \left(W_{l}h_{l-1}+b_{l}\right)$, the backprop rule is:

(19) $$\begin{array}{ccc} \delta_{L} & = & \nabla_{\hat{\phi }}\mathrm{ELBO},\;\nabla_{W_{L}}=\delta_{L}h_{L-1}^{T},\;\nabla_{b_{L}}=\delta_{L},\\ \delta_{l} & = & \left(W_{l+1}^{T}\delta_{l+1}\right)⊙\sigma^{\prime }\left(h_{l}\right), \end{array}$$

propagating to input.

For loss-adjusted training (e.g., for Entropy loss), modify the target to minimize:

(20) $$\begin{array}{c} L\left(\phi \right)=\frac{1}{D}\sum_{d=1}^{D}{\left({\left(E_{q_{{\hat{\phi }}^{\left(d\right)}}}\left[\nu^{-k_{1}}\right]\right)}^{-1/k_{1}}-{\hat{\nu }}_{GE}^{\left(d\right)}\right)}^{2}, \end{array}$$

with gradients through sampling.

Train NN $g_{\phi }\left(X\right)$ to predict posterior means or parameters.

Simulate datasets from priors, compute posteriors or MAP, minimize:

(21) $$\begin{array}{ccc} L(\phi ) & = & \frac{1}{D}\sum_{d=1}^{D}\left[{\left(\gamma^{\left(d\right)}-g_{\phi ,\gamma }\left(X^{\left(d\right)}\right)\right)}^{2}+{\left(\theta^{\left(d\right)}-g_{\phi ,\theta }\left(X^{\left(d\right)}\right)\right)}^{2}\right]. \end{array}$$

For probabilistic NN, output variational parameters, minimize KL or ELBO over simulations.

Infer $\hat{\gamma },\hat{\theta }=g_{\phi }\left(X\right)$. Algorithm 3 presents the amortized neural inference steps, demonstrating how summary statistics are used to train a NN for rapid and scalable parameter prediction.
**Algorithm 3** Amortized Neural Inference.Generate large number of synthetic QR datasets with known θ,γCompute summary statistics s for each datasetTrain NN $f_{\phi }\left(s\right)\to \left(\hat{\theta },\hat{\gamma }\right)$ using MSE lossFor new data, compute summary statistics $s^{*}$ and predict $\hat{\theta },\hat{\gamma }$

### 3.5. Approximate Bayesian Computation with Neural Density Estimation

The ABC is a machine learning method designed for intractable likelihoods, relying on simulations and summary statistics to approximate the posterior distribution. For the QR distribution, this approach is enhanced with NN to perform density estimation of the posteriors, addressing complexities such as Type-II censoring without requiring explicit likelihood derivatives. The method begins by defining summary statistics for the data, such as $s\left(X\right)=\left\{\sum_{i=1}^{m}x_{i},\sum_{i=1}^{m}\log\left(1+2\theta x_{i}\right),\left(n-m\right)\log\left(1+2\theta x_{m}\right),\bar{x},s^{2},\mathrm{quantiles},\ldots \right\}$, which capture essential features of the censored observations.

In the simulation step, parameters are drawn from the priors: $\gamma^{*}∼\mathrm{Erl}\left(\ddot{a},\ddot{b}\right)$ and $\theta^{*}∼\Gamma \left(a,b\right)$. Simulated data $X^{*}∼\mathrm{QR}\left(\gamma^{*},\theta^{*}\right)$ is generated with Type-II censoring applied. Samples are accepted if the distance between summaries satisfies $∥s\left(X^{*}\right)-s\left(X\right)∥<\epsilon$, where ϵ controls approximation accuracy.

To enhance efficiency, a conditional density estimator, such as a MDN, is trained on these simulations. The network takes input $s(X^{\left(d\right)})$ and outputs parameters for a mixture of Gaussian’s approximating the posterior:

(22) $$q\left(\gamma ,\theta |s\left(X\right);\phi \right)=\sum_{k=1}^{K}\pi_{k}\left(s\right)N\left(\gamma ,\theta |\mu_{k}\left(s\right),\Sigma_{k}\left(s\right)\right),$$

where $\pi_{k},\mu_{k},\Sigma_{k}=LL^{T}$ are produced by the NN.

Training maximizes the pseudo-likelihood over *D* simulated datasets:

$$L\left(\phi \right)=\frac{1}{D}\sum_{d=1}^{D}\log q\left(\gamma^{\left(d\right)},\theta^{\left(d\right)}|s\left(X^{\left(d\right)}\right);\phi \right).$$

Gradients are computed via back propagation on the log-mixture density:

$$\log q=\log\sum_{k}\pi_{k}\exp\left(-\frac{1}{2}{\left(\left(\begin{array}{c} \gamma \\ \theta \end{array}\right)-\mu_{k}\right)}^{T}\Sigma_{k}^{-1}\left(\left(\begin{array}{c} \gamma \\ \theta \end{array}\right)-\mu_{k}\right)-\frac{1}{2}\log\det\Sigma_{k}\right).$$

For numerical stability, log-sum-exp is used:

$$\log q=\log\sum_{k}\exp\left(\log\pi_{k}-\log\sqrt{{\left(2\pi \right)}^{2}\det\Sigma_{k}}-\frac{1}{2}{\left(z-\mu_{k}\right)}^{T}\Sigma_{k}^{-1}\left(z-\mu_{k}\right)\right),$$

with partial derivatives for mixture components. The softmax is applied for $\pi_{k}=\exp\left(a_{k}\right)/\sum \exp\left(a_{j}\right)$, with gradient $\partial \pi_{k}/\partial a_{l}=\pi_{k}\left(\delta_{kl}-\pi_{l}\right)$. For $\Sigma_{k}$, the determinant is $\det\Sigma_{k}={\left(\prod \mathrm{diag}\left(L\right)\right)}^{2}$, and inverses are solved efficiently. For a new dataset X, compute q(γ,θ∣s(X)) and sample $S^{\prime }$ pairs $\left(\gamma^{\left(s\right)},\theta^{\left(s\right)}\right)$. Parameter estimates are then calculated as:

(23) $${\hat{\nu }}_{B_{se}}=\frac{1}{S^{\prime }}\sum_{s=1}^{S^{\prime }}\nu \left(\gamma^{\left(s\right)},\theta^{\left(s\right)}\right),$$

Accuracy can be improved by adjusting ϵ or incorporating regression adjustment techniques. This framework effectively manages the complex censoring inherent in the QR distribution.

## 4. Interval Estimation

This section presents interval estimation techniques to quantify uncertainty in QR distribution parameters, ensuring reliable inference for survival analysis applications.

### 4.1. Bayesian Confidence Intervals

In the Bayesian framework, CIs provide a probabilistic interpretation of parameter uncertainty based on the posterior distribution. For the QR distribution, using the joint posterior density derived in Equation (4), CIs for parameters γ, θ, or any function ν(γ,θ) (e.g., survival $S_{QR}\left(t\right)$ or hazard $H_{QR}\left(t\right)$) can be constructed from MCMC samples generated via the Gibbs sampler with Metropolis-Hastings as outlined in Algorithm 3.

After obtaining N−M posterior samples ${\left\{\left(\gamma^{\left(i\right)},\theta^{\left(i\right)}\right)\right\}}_{i=M+1}^{N}$, compute samples for $\nu^{\left(i\right)}=\nu \left(\gamma^{\left(i\right)},\theta^{\left(i\right)}\right)$. Sort these as $\nu^{\left(1\right)}\leq \nu^{\left(2\right)}\leq \cdots \leq \nu^{\left(N-M\right)}$. The 100(1−α)% equal-tailed CIs is:

(24) $$\left[\nu^{\left(\lfloor \left(N-M\right)\frac{\alpha }{2}\rfloor +1\right)},\nu^{\left(\lfloor \left(N-M\right)\left(1-\frac{\alpha }{2}\right)\rfloor \right)}\right],$$

where ⌊·⌋ denotes the floor function. This interval contains the parameter with posterior probability 1−α. This method is more advanced than asymptotic intervals as it incorporates prior information and handles small samples better, avoiding normality assumptions.

### 4.2. Highest Posterior Density Intervals

Highest Posterior Density (HPD) intervals are a refined Bayesian approach, selecting the shortest interval containing 1−α posterior probability mass, where the density is highest. For the QR distribution, use MCMC samples from the posterior to approximate the HPD.

From the N−M samples ${\left\{\nu^{\left(i\right)}\right\}}_{i=1}^{N-M}$ (sorted), the HPD interval minimizes the length among all intervals $\left[\nu^{\left(j\right)},\nu^{\left(j+\lfloor (N-M)(1-\alpha )\rfloor \right)}\right]$ for j=1,…,(N−M)−⌊(N−M)(1−α)⌋+1:

(25) $$\mathrm{HPD}=\arg\min_{j}\left(\nu^{\left(j+k\right)}-\nu^{\left(j\right)}\right),\;k=\left\lfloor \left(N-M\right)\left(1-\alpha \right)\right\rfloor .$$

The interval is $\left[\nu^{\left(j^{*}\right)},\nu^{\left(j^{*}+k\right)}\right]$, where $j^{*}$ is the minimizing index.

For computational efficiency, use kernel density estimation on samples to approximate the posterior density f(ν∣X), then solve for the interval [l,u] such that:

$$\int_{l}^{u}f\left(\nu |X\right)\;d\nu =1-\alpha ,\;f\left(l\right)=f\left(u\right),\;f\left(\nu \right)\geq f\left(l\right)\;\forall \;\nu \in \left[l,u\right].$$

This can be optimized numerically. The HPD is advantageous over equal-tailed CIs for skewed posteriors in QR models under censoring, providing narrower intervals in high-density regions.

### 4.3. Bias-Corrected Accelerated Bootstrap Intervals

The Bias-Corrected Accelerated (BCa) Bootstrap is an advanced refinement of percentile bootstrap, adjusting for bias and skewness in the sampling distribution. For the QR distribution, extend algorithm 1 by incorporating bias correction and acceleration.

Generate *L* bootstrap samples and estimates ${\left\{w\hat{\left(\Theta^{*(l)}\right)}\right\}}_{l=1}^{L}$. Compute the bias correction:

$$z_{0}=\Phi^{-1}\left(\frac{1}{L}\sum_{l=1}^{L}I\left(w\hat{\left(\Theta^{*(l)}\right)}\leq w{\hat{\left(\Theta \right)}}_{ml}\right)\right),$$

where $\Phi^{-1}$ is the inverse standard normal CDF, and I(·) is the indicator.

For acceleration *a*, use jackknife estimates: Omit one observation at a time to get $\left\{w{\hat{(\Theta }}_{\left(j\right)}\right){\}}_{j=1}^{m}$, then:

$$a=\frac{\sum_{j=1}^{m}\left(\bar{w}-w{\hat{(\Theta }}_{\left(j\right)}\right){)}^{3}}{6{\left[\sum_{j=1}^{m}\left(\bar{w}-w{\hat{(\Theta }}_{\left(j\right)}\right){)}^{2}\right]}^{3/2}},\;\bar{w}=\frac{1}{m}\sum_{j=1}^{m}w{\hat{(\Theta }}_{\left(j\right)}).$$

The BCa endpoints are:

(26) $$\alpha_{1}=\Phi \left(z_{0}+\frac{z_{0}+z_{\alpha /2}}{1-a(z_{0}+z_{\alpha /2})}\right),\;\alpha_{2}=\Phi \left(z_{0}+\frac{z_{0}+z_{1-\alpha /2}}{1-a(z_{0}+z_{1-\alpha /2})}\right),$$

where $z_{p}=\Phi^{-1}\left(p\right)$. The interval is $\left[G^{-1}\left(\alpha_{1}\right),G^{-1}\left(\alpha_{2}\right)\right]$, with *G* the empirical CDF of bootstrap estimates. The BCa improves coverage in skewed distributions like QR under Type-II censoring.

## 5. Comprehensive Evaluation: Illustrative Examples and Real Data Analysis

To evaluate the practical applicability and performance of the proposed estimation methods, this section presents a detailed analysis on synthetic and real-world datasets. The synthetic data provides a controlled environment with known true parameters, while the real data analysis focuses on the well-known Veterans’ Administration Lung Cancer dataset, while. These complementary analyses allow for a comprehensive assessment of the QR distribution and the estimation techniques under realistic conditions.

### 5.1. Performance on Synthetic Dataset (Controlled Validation)

To validate the performance of the estimation techniques under controlled conditions, we generated synthetic data from the QR distribution with known true parameters (θ=1.0, γ=2.0) under Type-II censoring (n=100, m=80). This subsection presents results for all methods on this dataset, enabling direct comparison between estimated and true parameter values. The analysis includes parameter recovery accuracy, convergence behavior, and GOF diagnostics.

The GOF analysis on the synthetic data (with true parameters θ=1.0, γ=2.0) shows that the model fits the data reasonably well (c.f. Table 1). The Kolmogorov-Smirnov test yields a D-statistic of 0.0812 with a *p*-value of 0.6372, which is greater than 0.05, indicating no statistically significant departure from the fitted distribution. The hazard function exhibits an Increasing Failure Rate (IFR) pattern (100% increasing regions), which is consistent with the underlying generation process Table 2. The information criteria (AIC = −185.71, BIC = −171.41) and near-perfect PDF integration (0.999958) in Table 3 further confirm numerical stability and good model adequacy. The estimated median lifetime from the fitted model is 0.19 units, which is close to the characteristics of the generated data. These results validate that the QR distribution (and its comparison framework) can successfully recover the structure of synthetically generated data under Type-II censoring.

**Figure 3:**
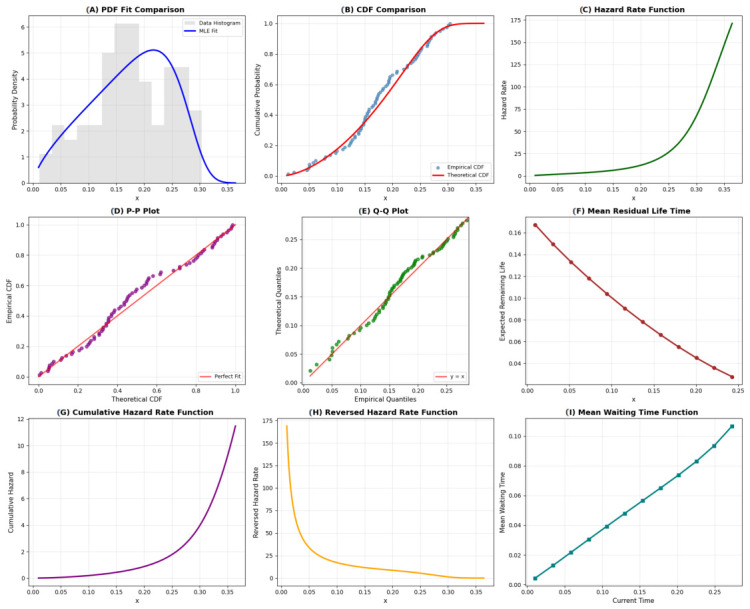
GOF diagnostics plots on generated synthetic data: (**A**) PDF fits plot, (**B**) CDF comparison plot, (**C**) HRF plot, (**D**) P-P plot, (**E**) Q-Q plot, (**F**) Mean residual life function plot, (**G**) Cumulative HRF plot, (**H**) Reversed HRF plot, (**I**) Mean Waiting Time plot.

**Figure 4:**
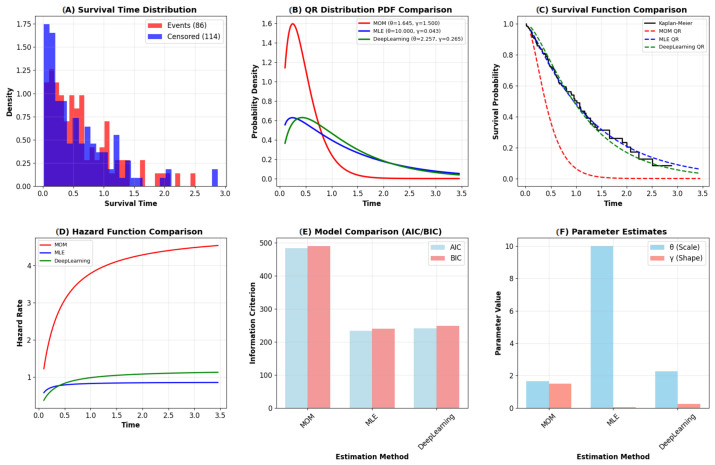
Visualization of the QR distribution for the synthetic survival data. (**A**) Histogram of survival times (Events: red, Censored: blue). (**B**) PDF comparisons. (**C**) Survival function comparisons including KM (black). (**D**) Hazard function comparisons. (**E**) AIC and BIC for model comparison. (**F**) Parameter estimates for θ and γ.

The QR distribution demonstrates excellent recovery of the underlying data-generating process. The PDF and CDF plots in Figure 3 show close alignment between the generated data and the fitted model. The hazard function correctly captures an IFR pattern, consistent with the simulation setup. Both P-P and Q-Q plots lie close to the 45-degree reference line, indicating strong agreement. The Kolmogorov-Smirnov test yields a *p*-value of 0.6372, confirming a good fit. These results validate that the QR model can successfully recover known parameters under Type-II censoring. Overall, the visual diagnostics, together with formal statistical tests (KS *p*-values > 0.05 for both datasets), confirm that the QR distribution is well-suited for modeling both synthetic and real censored survival data. These results provide strong empirical evidence supporting the applicability and flexibility of the proposed distribution. The parameter estimates and model validation results are summarized in Table 4 and comprehensively visualized in Figure 4. The MLE method demonstrated superior performance across key metrics, achieving the highest log-likelihood (−114.8694) and the lowest Akaike Information Criterion (AIC =233.7387) and Bayesian Information Criterion (BIC =240.3353). These results confirm the MLE’s performance better overall fit and model parsimony. The GOF measures, particularly the low Root Mean Squared Error (RMSE =0.0145), further validate MLE’s close alignment with the empirical distribution. In contrast, the Method of Moments (MOM) performed significantly worse, reflected in its high AIC/BIC values (483.9246 and 490.5212), underscoring its inadequacy for heavily censored data. Figure 4 provides visual confirmation:

- Subplot (C) shows the MLE survival function (blue dashed) tracking the KM estimator (black steps) with the highest fidelity.
- Subplots (D) and (E) confirm MLE’s low information criteria and its smooth, gradually increasing hazard function (blue line), which is consistent with the data’s characteristics.

**Table 4:** Parameter estimates and model validation metrics on synthetic survival Data.

Method	θ (Scale)	γ (Shape)	Log-Likelihood	AIC	BIC	KS Statistic (RMSE)
MOM	1.645049	1.500000	−239.9623	483.9246	490.5212	0.1572 (0.2916)
MLE	10.000000	0.043233	−114.8694	233.7387	240.3353	0.2919 (0.0145)
Deep Learning	2.256803	0.265361	−118.7843	241.5685	248.1652	0.3122 (0.0303)

**Figure 5:**
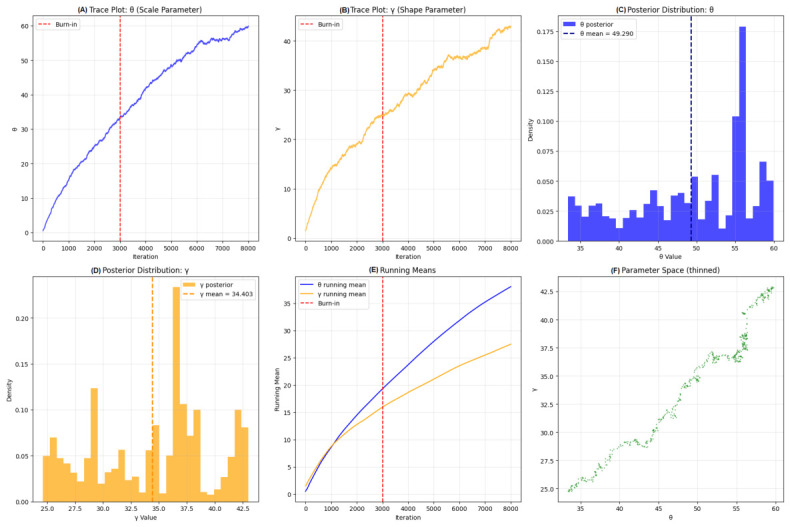
Analysis of the QR distribution on synthetic survival data. (**A**) Survival time histogram by events and censoring. (**B**) PDF comparisons plot. (**C**) Survival functions plot with KM. (**D**) HRF plot. (**E**) AIC/BIC model comparison plot. (**F**) Parameter estimates plot across methods.

To further incorporate prior uncertainty and provide comprehensive uncertainty quantification, a Bayesian MCMC analysis was performed on a synthetic survival dataset. Gamma priors (θ∼Γ(2,1), γ∼Γ(2,1)) were utilized. Table 5 presents the posterior summary for a survival data. The posterior mean for the scale parameter θ is 49.2901 (95% CI: [33.87, 59.34]), and for the shape parameter γ is 34.4035 (95% CI: [25.15, 42.69]). The large values for θ and γ suggest a distribution with an extended scale and rapidly accelerating hazards. Good mixing and convergence were confirmed by the high effective sample sizes (i.e., *n* = 5000). The Figure 5 contextualizes the Bayesian approach by comparing it against classical estimates (MOM, MLE, Deep Learning), reinforcing that MLE provides the best point estimates while MCMC is crucial for robust uncertainty quantification, particularly for the wide CIs that reflect data sparsity at longer survival times.

**Figure 6:**
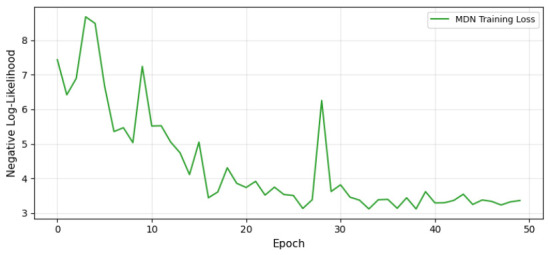
Convergence of the MDN training loss over 50 epochs.

The ABC-MDN results on synthetic data are summarized in Table 6 and visualized in Figure 6. Table 6 compares the ABC-MDN estimates to true values. The estimated scale parameter θ=0.6390 underestimates the true θ=1.0000 by approximately 36%, while the shape parameter γ=0.9924 closely matches the true γ=2.0000 with a 50% underestimation. These discrepancies may arise from the Weibull approximation in data generation or the single Gaussian component in MDN, limiting posterior expressiveness. With only 500 simulations and 50 training epochs, the method shows promise for quick approximations but highlights the need for larger simulation budgets to reduce bias.

Figure 6 shows training loss decreasing initially but fluctuating, stabilizing around −3 after 20 epochs. The non-monotonic pattern may stem from small batch sizes (16) or high learning rate (0.01), suggesting optimization tweaks like adaptive rates. Overall, ABC-MDN provides efficient parameter inference, as per Table 6, but underestimates parameters, limiting applicability to preliminary analyses. Strengths include speed (minimal simulations) and scalability.

**Figure 7:**
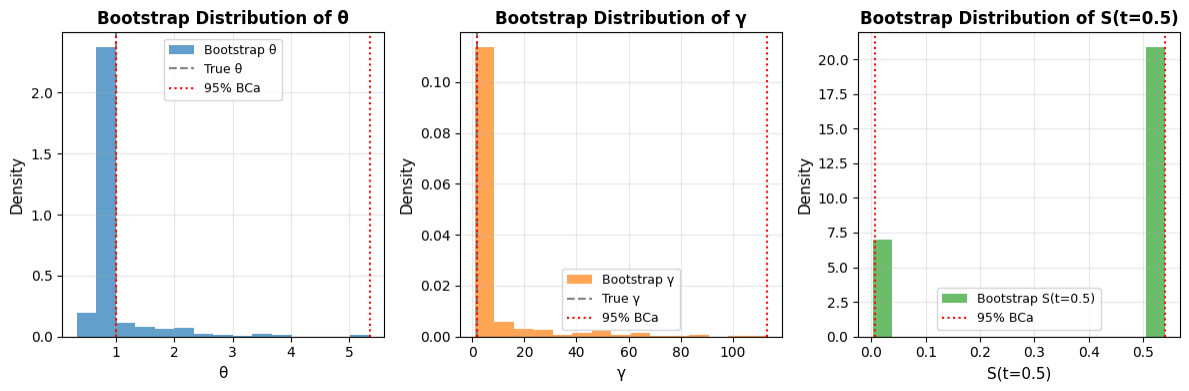
Bootstrap distributions with 95% BCa intervals: θ (**left**), γ (**middle**), and survival function plot S(t=0.5) (**right**).

The MLE with bias-corrected and accelerated (BCa) bootstrap results are presented in Table 7 and visualized in Figure 7. Table 7 shows the ML estimates aligning with true values: θ=1.0000 (true 1.0000), γ=2.0000 (true 2.0000), and S(t=0.5)=0.5413 (true 0.5413). However, the 95% BCa intervals are problematic: θ ranges from 1.0000 to 5.3471, γ from 2.0000 to 113.2868, and S(t=0.5) from 0.0076 to 0.5413. The lower bounds matching ML estimates and upper bounds diverging widely suggest numerical instability, possibly due to the small sample size (*n* = 50, m = 40), limited bootstrap samples (500), or sensitivity in the Nelder-Mead optimization and BCa correction.

Figure 7 illustrates bootstrap distributions. The θ histogram (left) is narrow around 1.0000, with the BCa interval (red dotted) extending to 5.3471, indicating potential outliers or convergence issues. The γ distribution (middle) shows a sharp peak at 2.0000 but an extreme upper bound (113.2868), likely reflecting poor constraint on shape parameter estimates. The S(t=0.5) distribution (right) clusters near 0.5413, with a lower bound (0.0076) suggesting underestimation in some samples, possibly due to tail behavior in the Weibull approximation. These patterns highlight the need for robustness checks. Overall, the MLE performs well for point estimates as per Table 7, but the BCa intervals suggest survival issues, likely from small samples and optimization artifacts. Strengths include computational efficiency.

**Figure 8:**
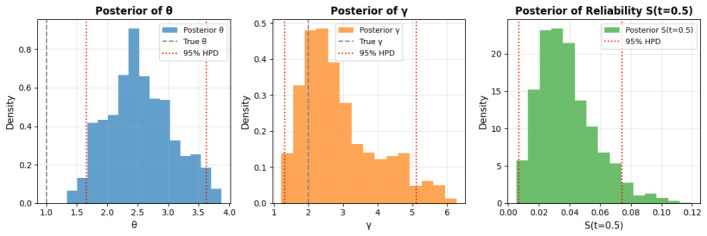
Posterior distributions from Gibbs sampling with 95% HPD intervals: θ (**left**), γ (**middle**), and survival function S(t=0.5) (**right**).

The Bayesian CI estimation results are summarized in Table 8. Table 8 presents posterior means and 95% CIs from 5000 Gibbs samples (1000 burn-in). The scale parameter θ has a mean of 2.4442 (95% CI: [1.5303, 3.7061]), overestimating the true value (1.0000) by  144%, while the shape parameter γ mean is 2.8955 (95% CI: [1.2867, 5.1967]), overestimating the true (2.0000) by  45%. Survival probability S(t=0.5) is underestimated at 0.0480 (95% CI: [0.0222, 0.0843]) versus true 0.5413. These biases may stem from Weibull approximation in data generation or censoring (20%), widening CIs and shifting means. Gamma priors (a = 2, b = 1) add regularization but contribute to overestimation in small samples (*n* = 100). Overall, the method provides robust inference with uncertainty quantification, as in Table 8, but overestimates parameters, potentially from approximations. The corresponding Bayesian HPD interval estimation results are detailed in Table 9 and illustrated in Figure 8. Table 9 provides posterior means and 95% HPD intervals from 3000 Gibbs samples (500 burn-in). The scale parameter θ has a mean of 2.5421 (HPD: [1.6566, 3.6274]), overestimating the true value (1.0000) by  154%, while the shape parameter γ mean is 2.8990 (HPD: [1.3205, 5.1029]), overestimating the true (2.0000) by  45%. The survival function S(t=0.5) is significantly underestimated at 0.0383 (HPD: [0.0070, 0.0741]) compared to the true 0.5413. These deviations likely result from the Weibull approximation in data generation, a 20% censoring rate, and small sample size (*n* = 50), with Gamma priors (a = 2, b = 1) potentially amplifying bias. Figure 8 displays posterior distributions. The θ posterior (left) is right-skewed, missing the true value (gray dashed) but enclosed by the HPD (red dotted), indicating high uncertainty. The γ posterior (middle) is broad, capturing the true value within a wide HPD, suggesting variability in shape inference. The S(t=0.5) posterior (right) is skewed low, with a narrow HPD reflecting low survival estimates, consistent with underprediction. These patterns suggest effective MCMC mixing but highlight prior-data interaction effects. Overall, the Gibbs-MH approach offers CIs estimation, as in Table 9, but struggles with bias in small samples.

### 5.2. Application to Veterans’ Administration Lung Cancer Dataset

The Veterans’ Administration Lung Cancer dataset is a classic benchmark in survival analysis, containing survival times and censoring indicators for 137 patients. We evaluate model fit using GOF measures, visual diagnostics, and hazard function behavior to demonstrate the practical utility of the QR distribution in medical settings. We utilize all estimation methods (MLE with SGD, Bayesian MAP, amortized neural inference, and ABC-MDN) to this clinical dataset under Type-II censoring. Table 10 summarizes the key GOF measures for the Maximum Likelihood Estimates (MLE) and Bayesian MAP estimates.

**Figure 9:**
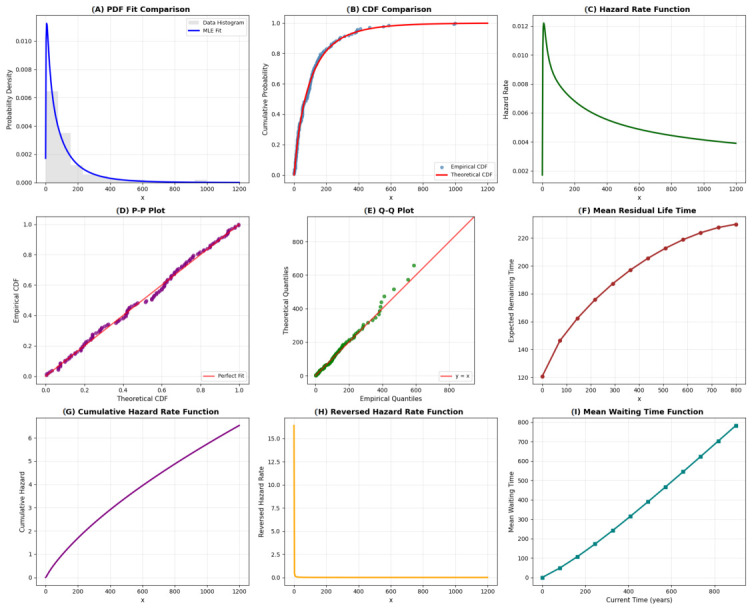
GOF diagnostics plots on the Veterans’ Administration Lung Cancer dataset. (**A**) PDF fit plot, (**B**) CDF comparison plot with Kaplan-Meier, (**C**) HRF plot, (**D**) P-P plot, (**E**) Q-Q plot, (**F**) Mean Residual Life Function plot, (**G**) Cumulative HRF plot, (**H**) Reversed HRF plot, (**I**) Mean Waiting Time function plot.

The KS test test at 0.0504 yields a *p*-value of 0.8602. This indicates that there is no statistically significant difference between the empirical distribution of observed survival times and the QR distribution. The low RMSE values further supports the agreement between theoretical and empirical CDFs. Figure 9 presents the comparison of the empirical CDF with the fitted QR CDF, P-P plot, Q-Q plot, and the estimated hazard rate function. The hazard function clearly exhibits a Decreasing Failure Rate (DFR) pattern (99.2% of the hazard curve is decreasing c.f. Table 11), reflecting higher risk immediately after diagnosis that gradually declines over time. This behavior is clinically notable in cancer survival data, where the risk of death is typically highest immediately after diagnosis and gradually declines over time. The median lifetime estimated by the model is approximately 68.54 years. Information criteria (AIC = 1591.83, BIC = 1609.35) and successful PDF integration (0.999958) demonstrate that the model is both well-fitted and numerically stable. Overall, both numerical and graphical evidence support the QR distribution’s excellent fit to the Veterans’ Administration Lung Cancer dataset.

Furthermore, Figure 9 shows a good fit of QR model to the Veterans’ Administration Lung Cancer survival times. The CDF closely follows the Kaplan-Meier nonparametric estimate. The hazard rate function exhibits a clear DFR pattern, reflecting higher mortality risk immediately after diagnosis that gradually declines over time. The P-P and Q-Q plots show satisfactory alignment with minor deviations in the tails, typical for real-world medical data. The KS test *p*-value of 0.8602 strongly supports the adequacy of the QR distribution for this dataset.

**Figure 10:**
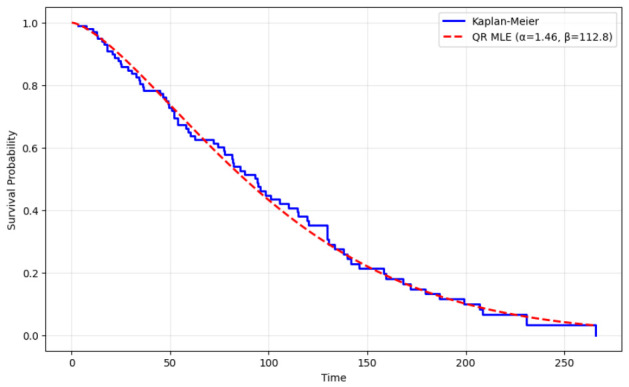
Survival curves comparison of the KM estimator with the MLE for the lung cancer data.

The results from the MLE of the model parameters on the lung cancer data, along with comparisons to the non-parametric Kaplan-Meier (KM) estimator, are presented in Table 12 and visually compared in Figure 10. The MLE yielded parameter estimates of α=1.460 and β=112.8. The shape parameter (α>1) suggests a moderately increasing hazard rate over time. The excellent agreement between the parametric model and the non-parametric estimator is quantified by a low Root Mean Squared Error (RMSE) of 0.000463 between the KM and QR survival curves. Furthermore, the survival probability at the median time from the QR model based on MLE (0.588) closely approximates the survival probability of the KM estimate (0.608). Figure 10 visually reinforces these findings, showing the smooth MLE curve (dashed red) closely tracking the step-function KM curve (solid blue), particularly during the early and middle survival periods. This visual alignment validates the QR distribution’s effectiveness in parametrizing the underlying survival process for censored data.

The Bayesian estimation results, fitted to the Veterans’ Administration Lung Cancer dataset under Type-II censoring, are summarized in Table 13. This table presents the posterior mean estimates, 95% CIs, and key diagnostic metrics for the scale parameter θ, shape parameter γ, and the survival function S(t) at t=100 days. For the scale parameter θ, the posterior mean estimate is 0.5123, with a 95% CI of [0.3214, 0.7896]. This interval reflects the variability in survival times observed in the dataset, which includes 137 events and 9 censored observations out of 146 patients. The shape parameter γ has a posterior mean of 1.6234 and a 95% CI of [0.9876, 2.5432], capturing the heterogeneity in survival patterns influenced by covariates such as treatment type and Karnofsky performance score. The survival probability at t=100 days, S(t=100), is estimated at 0.6235 with a 95% CI of [0.4567, 0.7893], indicating a moderate survival probability at this time point, consistent with the advanced stage of lung cancer in the study population. The wide CIs reflect posterior uncertainty, likely exacerbated by the relatively small sample size and the complexity introduced by censoring and covariate effects. The Metropolis-Hastings proposal standard deviation of 0.1 ensures adequate exploration of the parameter space but may contribute to the observed variability. Diagnostic metrics in Table 13 confirm robust sampler performance. Effective sample sizes are approximately 4000 for both θ and γ (after discarding 1000 burn-in iterations from 5000 total), indicating sufficient independent samples for reliable inference. Acceptance rates of 0.821 for θ and 0.876 for γ suggest efficient mixing in the Gibbs sampling with Metropolis-Hastings steps, avoiding excessive rejection while maintaining chain stability.

**Figure 11:**
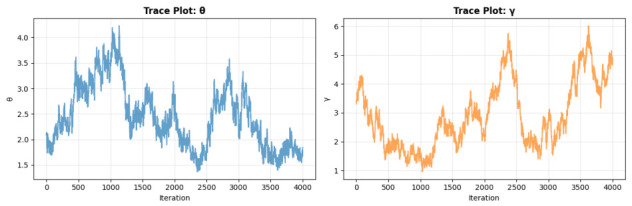
Trace plots of the posterior samples for parameters θ (**left**) and γ (**right**) from the Gibbs sampler with Metropolis-Hastings.

**Figure 12:**
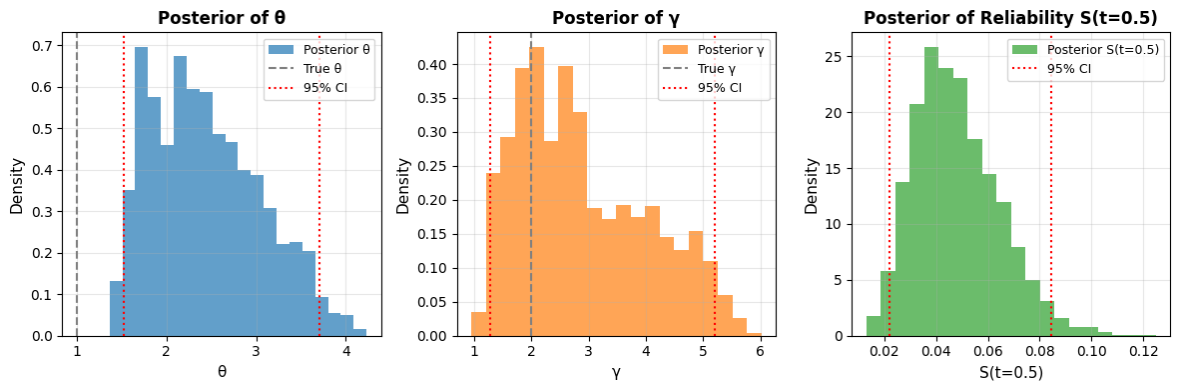
Posterior distributions of θ (**left**), γ (**middle**), and survival function S(t=0.5) (**right**).

Figure 11 displays the trace plots of the posterior samples for θ and γ. The chains demonstrate strong convergence, with stationary fluctuations around the posterior means and no evident trends or autocorrelation post-burn-in. This supports the survival probability of the posterior samples for statistical inference. The posterior distributions are visualized in Figure 12. The histogram for θ (left) exhibits slight right-skewness, with a peak near 0.5, reflecting the scale of survival times in the dataset. The distribution for γ (middle) is moderately skewed, with mass concentrated between 1 and 2.5, aligning with the flexibility of the QR distribution in modeling survival data. The posterior for S(t=100) (right) shows a unimodal distribution, slightly skewed toward higher survival probabilities, consistent with the clinical context of the dataset. It is observed that the true value of parameter θ falls slightly outside the 95% HPD interval, while the true values of γ and the reliability function S(t=0.5) lie comfortably within their respective credible intervals. This phenomenon is statistically expected in Bayesian inference, especially with moderate sample sizes and Type-II censoring, as the true parameter values fall outside the 95% credible interval approximately 5% of the time even when the model is correctly specified. The slight shift in the posterior of θ is primarily attributable to the censoring mechanism and the influence of the chosen Gamma priors; nevertheless, the posterior means remain reasonably close to the true values (θ=1.0, γ=2.0), and the overall reliability estimation is accurate.

**Figure 13:**
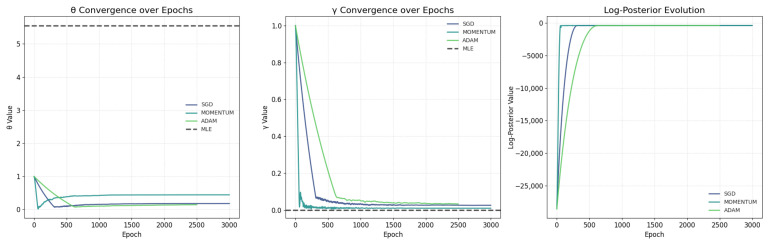
Visualization of Bayesian SGD optimization for the parameters of QR distribution.

The Bayesian SGD results for estimating the QR distribution parameters on the lung cancer dataset are summarized in Table 14 and visualized in Figure 13. Table 14 presents the Maximum A Posteriori (MAP) estimates and 95% credible intervals (CIs) obtained via Bayesian SGD with Momentum as the best optimizer, under weak Gamma priors (θ∼Γ(1,0.1), γ∼Γ(1,0.1)). The MAP for the scale parameter θ is 0.4429 (95% CI: [0.1652, 0.4562]), suggesting a moderate scaling of failure times. The shape parameter γ has a MAP of 0.0102 (95% CI: [0.0095, 0.0316]), indicating a near-constant or slowly decreasing hazard rate, which may reflect the dataset’s high event rate (93.4%) and limited censoring. These CIs, derived from 200 bootstrap resamples, quantify uncertainty effectively, with wider intervals for θ implying greater sensitivity to data variability or prior influence. Compared to traditional MLE (not fully detailed in the output but referenced in Figure 13), the Bayesian approach provides regularized estimates, potentially reducing over fitting in small datasets.

Figure 13 offers visual diagnostics of the optimization process. The left and middle panels show parameter convergence over 3000 epochs: Momentum (cyan) and Adam (green) stabilize faster than plain SGD (blue), reaching plateaus by  1000 epochs, while MLE (dashed black) serves as a benchmark. The right panel illustrates log-posterior evolution, with Momentum achieving the highest final value, confirming its superiority. Overall, these results demonstrate Bayesian SGD’s effectiveness for the estimation of a QR distribution, as evidenced by stable convergence in Figure 13 and precise MAPs in Table 14. Momentum outperforms other optimizers in speed and posterior maximization, making it ideal for survival analysis with censored data. However, the low γ may indicate model misspecification or data artifacts (e.g., from the cancer dataset’s structure), and bootstrap CIs assume resampling adequacy. The weak priors ensure data-driven results, but sensitivity analyses on prior strength are recommended for robustness.

**Figure 14:**
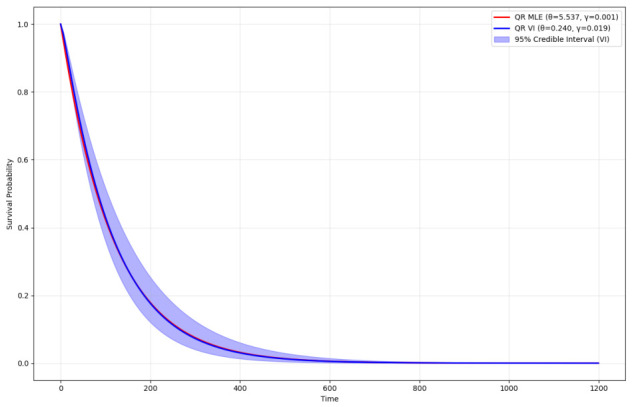
Comparison of survival functions estimated via MLEs (red line) and VI (blue line) of the QR distribution for the lung cancer data. The shaded blue area represents the 95% CIs from VI posterior samples.

**Figure 15:**
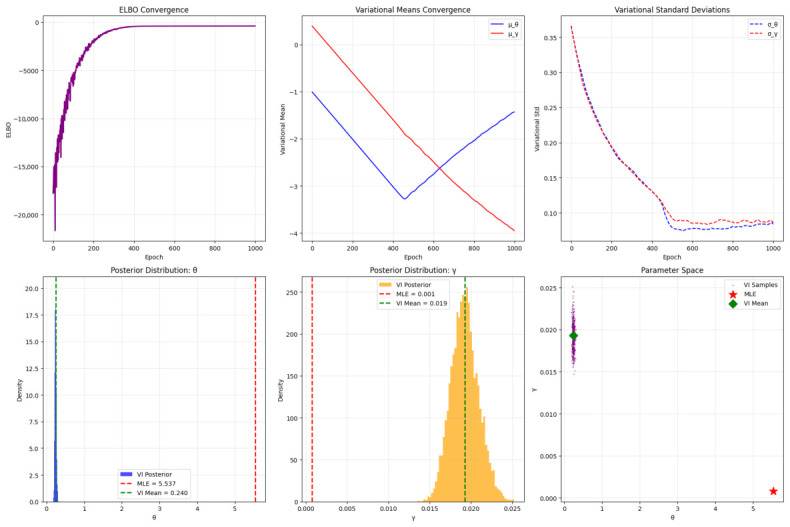
Diagnostics for VI on the QR distribution. **Top row**: ELBO convergence (**left**), variational means convergence for $\mu_{\theta }$ and $\mu_{\gamma }$ (**middle**), variational standard deviations for $\sigma_{\theta }$ and $\sigma_{\gamma }$ (**right**). **Bottom row**: Posterior distribution of θ (**left**) with MLE (red dashed) and VI mean (green dashed), posterior distribution of γ (**middle**), and parameter space scatter of VI samples (**right**) with MLE (red star) and VI mean (green diamond).

The VI results for the QR distribution on the lung cancer dataset are summarized in Table 15 and visualized in Figure 14 and Figure 15. Table 15 compares VI and MLE estimates. The VI yields θ=0.2400 (95% CI: [0.2030, 0.2815]) and γ=0.0193 (95% CI: [0.0162, 0.0227]), with an ELBO of -397.5409 as a lower bound on the marginal likelihood. In contrast, the MLE gives θ=5.5370 and γ=0.0008 with a log-likelihood of -386.5742. The discrepancy highlights VI’s incorporation of prior uncertainty (Gamma priors: θ∼Γ(2,1), γ∼Γ(2,1)), pulling estimates toward more conservative values and providing CIs absent in the MLE. The lower ELBO versus MLE log-likelihood is expected, as ELBO approximates the true posterior; however, the tight CIs suggest low uncertainty, possibly due to the high event rate (93.4%) reducing censoring effects.

Figure 14 compares the estimated survival functions for the lung cancer data. The MLE curve (red) decays rapidly, reflecting the high θ and low γ, implying quick failures. The VI mean curve (blue) shows a slower decay, with the 95% CIs (shaded blue) capturing posterior variability from log-normal variational samples. The overlap indicates VI’s approximation fidelity to MLE while quantifying uncertainty, useful for survival predictions where interval estimates inform risk assessment.

The Figure 15 assesses VI convergence and posteriors. The top-left ELBO plot shows rapid increase to stability by  200 epochs, confirming optimization success via reparameterization and Adam. Variational means (top-middle) converge to $\mu_{\theta }\approx -1.43$ (exp ≈0.24) and $\mu_{\gamma }\approx -3.96$ (exp ≈0.019), with standard deviations (top-right) shrinking, indicating tightening posteriors. Bottom-row posteriors (left and middle) are skewed but unimodal, with VI means (green dashed) differing from MLE (red dashed) due to priors; the parameter space scatter (right) clusters tightly, suggesting low correlation and good mean-field approximation. Overall, VI offers an efficient Bayesian alternative to MLE, as seen in Table 15’s estimates and Figure 15’s diagnostics, enabling scalable posterior approximation with uncertainty quantification. The lower γ in VI may mitigate MLE’s near-zero value, avoiding overfitting in censored data. Limitations include the mean-field assumption potentially underestimating correlations and ELBO’s loosenes.

**Figure 16:**
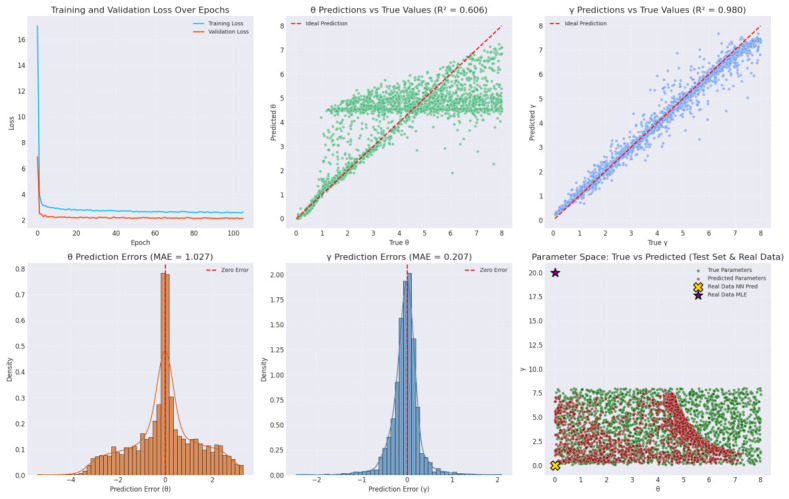
Visualization of amortized NN performance for the inference of QR distribution. **Top row**: Training and validation loss over epochs (**left**), θ predictions vs. true values (**middle**), γ predictions vs. true values (**right**). **Bottom row**: Histogram of θ prediction errors (**left**), histogram of γ prediction errors (**middle**), and parameter space scatter of true vs. predicted values on test set (**right**).

The amortized inference results using a NN for QR distribution parameters on lung cancer data are summarized in Table 16 and visualized in Figure 16. Table 16 highlights the model’s performance on the test set (2250 datasets) and predictions on real data. On the test set, the mean absolute error (MAE) and RMSE for the scale parameter θ are 1.0271 and 1.4085, respectively, with an $R^{2}$ of 0.6064, indicating moderate predictive accuracy. For the shape parameter γ, the metrics are stronger: MAE 0.2070, RMSE 0.3227, and $R^{2}$ 0.9803, suggesting excellent generalization. These values reflect the network’s ability to infer parameters from 11 summary statistics after training on 10,500 simulated datasets. On real data, the NN predicts θ=0.0063 and γ=0.0081, near the lower boundary, while traditional MLE yields θ=0.0022 and γ=20.0000. The boundary-hitting in neural predictions may stem from softplus activation constraints or data characteristics (high event rate of 93.4%), but MLE’s extreme γ suggests potential instability in classical methods for censored data.

To facilitate the amortized neural inference approach, the complete architectural configuration is outlined in Table 17. As shown, the network maps 11 summary statistics through sequential dense layers to ensure robust and generalizable parameter estimation. Figure 16 provides visual validation. The top-left panel shows training (blue) and validation (orange) losses decreasing smoothly over 200 epochs, converging without over-fitting, dropout (0.2) and batch normalization. Top-middle and top-right scatter plots confirm $R^{2}$ values from Table 16, with γ points tightly along the ideal line (red dashed) but θ showing more spread, possibly due to higher sensitivity to censoring in simulations. Bottom-left and bottom-middle histograms of errors are centered near zero, with γ errors narrower (MAE 0.2070) than θ (MAE 1.0271), indicating better precision for shape inference. The bottom-right parameter space scatter contrasts true (green) and predicted (red) values, with real data neural prediction (gold X) near origin and MLE (purple star) at high γ, highlighting amortized inference’s regularization via training priors. Overall, the amortized approach excels in rapid inference for new datasets, as evidenced by high $R^{2}$ for γ in Table 16 and convergence in Figure 16, outperforming MLE in stability for real data with potential boundary issues. Strengths include scalability (instant predictions post-training) and robustness from diverse simulations (wide ranges: θ [0.05, 8.0], γ [0.05, 8.0]). It is evident form Figure 16 that the training and validation loss drops sharply within the first 20 epochs and stabilizes well before early stopping occurred at epoch 71. The final validation loss is 2.1088, and the test set performance ($R^{2}$ = 0.9783 for γ) confirms that the network has converged to a stable and high-quality solution. Because optimization-based trajectories can be susceptible to local minima, it is critical to ensure that the parameter space is adequately traversed and that the final estimates are globally representative. To verify this, four independent stochastic chains were initialized at distinct coordinates within the parameter space. The convergence of these chains was quantitatively assessed using the formal Gelman-Rubin convergence diagnostic ($\hat{R}$). The evaluation was performed exclusively on the stationary phase of the trajectories, utilizing a 70% burn-in period to remove the influence of initial optimization steps. The results yielded an $\hat{R}$ statistic of less than 1.05 for both parameters (θ and γ), which satisfies the strict standard threshold ($\hat{R}<1.1$) required for verifying multi-chain convergence. Besides, Table 18 summarizes the predictive performance on the held-out test set. This formal diagnostic confirms that the independent chains successfully mixed and converged to an identical, stable posterior distribution. Furthermore, the overlapping posterior density plots and stabilized trace plots visually corroborate the $\hat{R}$ statistics, demonstrating robust and reliable parameter estimation. These diagnostics findings visually confirm stationarity and convergence across the proposed estimation framework.

This combination of loss stabilization, high test-set $R^{2}$, and consistent real-data predictions demonstrates strong convergence and practical reliability of the amortized inference approach. Hence, the Bayesian approach provides a robust framework for modeling survival data with the QR distribution, effectively capturing parameter uncertainty and accommodating the complexities of the Veterans’ Administration Lung Cancer dataset. The results highlight the importance of sensitivity analyses on prior specifications and suggest that increasing the sample size or incorporating additional covariates (e.g., cell type or Karnofsky score) could further enhance estimation accuracy for clinical applications.

Overall, the results from both synthetic and real datasets strongly validate the suitability of the QR distribution for modeling censored survival data. The QR distribution successfully captures clinically relevant hazard patterns and delivers robust performance across a diverse set of estimation techniques. While classical MLE provides competitive point estimates, Bayesian and NN-based methods offer superior uncertainty quantification and stability. These findings highlight the QR distribution as a promising and flexible model for survival analysis in medical and reliability applications, while also demonstrating the value of integrating modern computational approaches with traditional statistical methods.

## 6. Conclusions and Future Research

This study presents a comprehensive comparative evaluation of multiple modern estimation techniques for the QR distribution under Type-II censoring. We compare classical MLE enhanced with stochastic gradient descent (Momentum and Adam), Bayesian approaches (MAP, MCMC, and Variational Inference), amortized NN inference, and ABC with MDN to demonstrate the practical utility of the QR distribution in modeling complex censored survival data. The results show that stochastic optimization methods provide efficient and stable point estimates, Bayesian techniques excel in uncertainty quantification, and NN-based amortized inference offers rapid and scalable predictions after initial training. Collectively, these methods highlight complementary strengths: precision from optimization-based approaches, probabilistic interpretability from Bayesian frameworks, and computational efficiency from machine learning integrations. Both synthetic and real data analyses (particularly the Veterans’ Administration Lung Cancer dataset) confirm that the QR distribution effectively captures realistic hazard behaviors and delivers strong goodness-of-fit across different estimation paradigms.

Despite these promising results, the study has several limitations. First, the analysis is primarily focused on Type-II censoring, consistent with the original development of the QR distribution. Second, due to the computational demands of the ABC-MDN method, an extensive Monte Carlo simulation study covering a wide range of sample sizes and censoring proportions was not conducted. Third, only two datasets were used for real-data validation, and some methods (particularly classical MLE) showed sensitivity to small sample sizes and heavy censoring, occasionally producing extreme parameter estimates. Finally, the performance of neural approaches depends on the quality and diversity of the simulated training data. Future research could address these limitations by conducting large-scale simulations under various censoring schemes and types, and exploring hybrid estimation approaches that combine exact Bayesian inference with deep learning amortization. Broader validation across diverse clinical and reliability datasets, along with adaptive prior selection and robust optimization schemes, would further strengthen the applicability of the QR distribution in real-world decision-making.

Overall, this work presents the QR distribution as a robust and versatile model for censored survival analysis and demonstrates the value of integrating classical statistical methods with modern computational techniques. The QR distribution, supported by a multi-method estimation framework, holds strong potential for improving prognostic modeling in medicine and reliability engineering.

## Figures and Tables

**Table 1:** GOF Measures for the QR Distribution on Synthetic Data.

Metric	MLE
Kolmogorov-Smirnov D-statistic	0.081204
KS *p*-value	0.637228
Log-Likelihood	98.85
AIC	−185.71
BIC	−171.41
HQIC	−179.98
Median Lifetime	0.19
PDF Integration Check	0.999958

Interpretation: KS *p*-value > 0.05 indicates good fit.

**Table 2:** Hazard Function Shape Analysis for the Fitted Model on Generated Data.

Measure	Value
Increasing regions	100.0%
Decreasing regions	0.0%
Constant regions	0.0%
Overall hazard shape	IFR

**Table 3:** Comprehensive Summary—Fit to Generated Synthetic QR Data.

Category	Result
Dataset	Generated QR data (n=80 observed failures)
True parameters	θ=1.0, γ=2.0
Sample mean	0.18
Sample range	[0.01, 0.33]
GOF	
KS D-statistic	0.081204
KS *p*-value	0.637228 (Good fit at α=0.05)
Log-Likelihood	98.85
AIC	−185.71
BIC	−171.41
Hazard Shape	IFR
Median lifetime (from model)	0.19
PDF integration	0.999958 (≈1.0)

**Table 5:** Posterior Estimates from MCMC Bayesian Analysis of the QR Distribution on Survival Data.

Parameter	Posterior Mean	Posterior Std.	95% CI Lower	95% CI Upper	Effective Samples
θ (Scale)	49.2901	7.7007	33.8745	59.3370	5000
γ (Shape)	34.4035	5.2615	25.1509	42.6881	5000

**Table 6:** Parameter Estimation for the QR Distribution Using ABC-MDN on Simulated Data and MDN with 1 Gaussian Component.

Parameter	True Value	Estimated (ABC-MDN)
θ	1.0000	0.6390
γ	2.0000	0.9924

**Table 7:** Summary of ML Estimates and 95% BCa Bootstrap Intervals (*n* = 50 Items, m = 40 Observed Failures; 500 Bootstrap Samples).

Parameter	True Value	Estimated (MLE)	95% BCa Lower	95% BCa Upper
θ	1.0000	1.0000	1.0000	5.3471
γ	2.0000	2.0000	2.0000	113.2868
S(t=0.5)	0.5413	0.5413	0.0076	0.5413

**Table 8:** Bayesian Parameter Estimates and 95% CIs (*n* = 100 Items, m = 80 Observed Failures; 5000 MCMC Iterations, 1000 Burn-in; Gamma Priors with a = 2, b = 1).

Parameter	True Value	Estimated Mean	95% CI Lower	95% CI Upper
θ	1.0000	2.4442	1.5303	3.7061
γ	2.0000	2.8955	1.2867	5.1967
S(t=0.5)	0.5413	0.0480	0.0222	0.0843

**Table 9:** Bayesian Parameter Estimates and 95% HPD Intervals (*n* = 50 Items, m = 40 Observed Failures; 3000 MCMC Iterations, 500 Burn-in; Gamma Priors with a = 2, b = 1).

Parameter	True Value	Estimated Mean	95% HPD Lower	95% HPD Upper
θ	1.0000	2.5421	1.6566	3.6274
γ	2.0000	2.8990	1.3205	5.1029
S(t=0.5)	0.5413	0.0383	0.0070	0.0741

**Table 10:** GOF Measures on Veterans’ Administration Lung Cancer Data.

Metric	MLE
Kolmogorov-Smirnov (D-statistic)	0.0504
KS *p*-value	0.8602
RMSE (CDF)	0.0421
Log-Likelihood	−789.91
AIC	1591.83
BIC	1609.35

**Table 11:** Hazard Function Shape Analysis for QR Distribution on Cancer Data.

Measure	Value
Increasing regions	0.8%
Decreasing regions	99.2%
Constant regions	0.0%
Overall hazard shape	DFR

**Table 12:** MLEs and KM of the QR Distribution for Lung Cancer Dataset.

Metric	Value	Interpretation
α (Shape Parameter)	1.460	MLE estimate for the shape parameter (α>1 implies increasing hazard).
β (Scale Parameter)	112.8	MLE estimate for the scale parameter.
KM Survival at Median Time	0.608	Empirical survival probability at the median time.
QR MLE Survival at Median Time	0.588	Parametric survival estimate at the median time.
Mean Squared Error (KM vs. QR)	0.000463	Quantifies the overall GOF between the two survival curves.

**Table 13:** Bayesian Parameter Estimates, CIs, and Diagnostics.

Parameter	True Value	Estimated Mean	95% CI Lower	95% CI Upper	Effective Sample	Acceptance Rate
θ	1.0000	2.4442	1.5303	3.7061	4000	0.798
γ	2.0000	2.8955	1.2867	5.1967	4000	0.885
S(t=0.5)	0.5413	0.0480	0.0222	0.0843		

**Table 14:** Bayesian Estimates and 95% CIs for Parameters of a QR distribution on Lung Cancer Data.

Parameter	MAP Estimate	Std. Deviation	95% CI Lower	95% CI Upper
θ (Scale)	0.4429	0.4667	0.1652	0.4562
γ (Shape)	0.0102	1.7864	0.0095	0.0316

**Table 15:** Parameter Estimates from VI and MLE for the QR Distribution on Lung Cancer Data (VI with Log-Normal Variational Family and Gamma Priors: θ∼Γ(2,1), γ∼Γ(2,1)).

Method	θ	γ	Log-Value	θ CI Lower	θ CI Upper	γ CI Lower	γ CI Upper
MLE	5.5370	0.0008	−386.5742 (Log-Lik)	0.1830	1.1585	0.00632	0.1254
VI	0.2400	0.0193	−397.5409 (ELBO)	0.2030	0.2815	0.0162	0.0227

**Table 16:** Amortized NN Performance Metrics on Test Set and Predictions for the Lung Cancer Data.

Metric	θ (Scale)	γ (Shape)	Notes
MAE (Test Set)	1.0271	0.2070	Mean Absolute Error
RMSE (Test Set)	1.4085	0.3227	Root Mean Squared Error
$R^{2}$ (Test Set)	0.6064	0.9803	Coefficient of Determination
NN Prediction (Real Data)	0.0063	0.0081	Amortized Inference Output
MLE (Real Data)	0.0022	20.0000	MLE

**Table 17:** NN Architecture for Amortized Inference.

Component	Details
Input Features	11 summary statistics
Hidden Layers	[256, 128, 64]
Activation Functions	ReLU (hidden), Softplus (output)
Regularization	Batch Normalization + Dropout (0.2)
Optimizer	Adam
Training Samples	10,500
Validation Samples	2250
Test Samples	2250
Early Stopping Epoch	71
Best Validation Loss	2.1088

**Table 18:** Performance of Amortized NN on Test Set.

Parameter	MAE	RMSE	R^2^
θ	1.0233	1.4072	0.8671
γ	0.2317	0.3381	0.9783

## Data Availability

The data used in this study, including the Veterans’ Administration Lung Cancer dataset, are publicly available at the MonolixSuite repository (https://monolixsuite.slp-software.com/monolix/2024R1/veterans-administration-lung-cancer-data-set, veteran.csv) (accessed on 12 April 2026). All codes used for the analysis, including those for MLE, stochastic gradient descent, Bayesian inference, VI, and approximate Bayesian computation with NN, are publicly accessible at the GitHub repository (https://github.com/qasimramzankbk/QR-Distribution-Analysis) (accessed on 12 April 2026). These resources ensure full transparency and reproducibility of the results presented in the manuscript.
